# CRISPR-DIPOFF: an interpretable deep learning approach for CRISPR Cas-9 off-target prediction

**DOI:** 10.1093/bib/bbad530

**Published:** 2024-02-08

**Authors:** Md Toufikuzzaman, Md Abul Hassan Samee, M Sohel Rahman

**Affiliations:** Department of Computer Science and Engineering, Bangladesh University of Engineering and Technology, Dhaka, 1205, Bangladesh; Department of Integrative Physiology, Baylor College of Medicine, Houston, TX 77030, USA; Department of Computer Science and Engineering, Bangladesh University of Engineering and Technology, Dhaka, 1205, Bangladesh

**Keywords:** CRISPR Cas-9, Off-Target, LSTM, Interpretation, Genetic Algorithm, Transformers

## Abstract

CRISPR Cas-9 is a groundbreaking genome-editing tool that harnesses bacterial defense systems to alter DNA sequences accurately. This innovative technology holds vast promise in multiple domains like biotechnology, agriculture and medicine. However, such power does not come without its own peril, and one such issue is the potential for unintended modifications (Off-Target), which highlights the need for accurate prediction and mitigation strategies. Though previous studies have demonstrated improvement in Off-Target prediction capability with the application of deep learning, they often struggle with the precision-recall trade-off, limiting their effectiveness and do not provide proper interpretation of the complex decision-making process of their models. To address these limitations, we have thoroughly explored deep learning networks, particularly the recurrent neural network based models, leveraging their established success in handling sequence data. Furthermore, we have employed genetic algorithm for hyperparameter tuning to optimize these models’ performance. The results from our experiments demonstrate significant performance improvement compared with the current state-of-the-art in Off-Target prediction, highlighting the efficacy of our approach. Furthermore, leveraging the power of the integrated gradient method, we make an effort to interpret our models resulting in a detailed analysis and understanding of the underlying factors that contribute to Off-Target predictions, in particular the presence of two sub-regions in the seed region of single guide RNA which extends the established biological hypothesis of Off-Target effects. To the best of our knowledge, our model can be considered as the first model combining high efficacy, interpretability and a desirable balance between precision and recall.

## INTRODUCTION

CRISPR Cas-9 stands for Clustered Regularly Interspaced Short Palindromic Repeats and CRISPR-associated enzyme Cas-9. It utilizes a bacterial defense mechanism to precisely cut DNA sequences, which has been repurposed by scientists to cause a double-strand break at any portion of the DNA by providing the Cas-9 enzyme with the exact sequence of that DNA portion [1]. After the break, DNA’s natural healing mechanism is triggered, and due to mutation, the repair region’s biological function gets disrupted. Thus, the CRISPR Cas-9 system can be exploited to knock off specific genes in an organism’s genome. This repair mechanism can be controlled by providing a template to replace the existing DNA sequence [2, 3], and thus the gene gets edited.

The applications of CRISPR are beyond imagination. CRISPR can be used to map the functionality of genes in different organisms. It can revolutionize the agriculture sector by designing disease and weed-resistant high-yielding crops [4, 5]. It can also be used for curing genetic diseases [5]. However, like any other groundbreaking advancement, CRISPR comes with its own set of challenges, particularly in the form of Off-Target effects where the single guide RNA (sgRNA) causes a double-strand break of the DNA even with the presence of a few nucleotide mismatches. Identifying these Off-Targets through traditional laboratory validation methods can be costly and time-consuming. This presents a valuable opportunity for computational methods to offer a rapid, reliable and cost-effective solution to detect and predict Off-Target effects in genome-editing.

The detection of potential Off-Target effects is very crucial for the successful application of the CRISPR Cas-9 system. In recent years, computational approaches, especially machine learning-based ones, have contributed to a great extent in fields like genomic sequence analysis, drug discovery, motif finding, etc. [6]. Following the same trend, computational approaches have also been used to predict the Off-Target effects [7]. The early detection methods relied on score-based approaches [8–11] primarily focusing on identifying Off-Target effects based on the presence and positions of mismatches between the target site and the guide RNA assigning scores based on the mismatches. These methods do not consider the complex relationship among the sequences, and they may suffer from experimental variations and limitations [12].

Traditional machine learning approaches for Off-Target prediction typically involve the use of manually engineered features. These features can include sequence-based characteristics, such as mismatch patterns or nucleotide composition, and contextual information surrounding the target site. Abadi *et al*. [13] aggregated datasets from three different sources: GUIDE-Seq [14], HTGTS [15] and BLESS [16], and trained a random forest regression model. Peng *et al*. [17] trained several support vector machine (SVM) models and ensembled them where the number of positive samples and negative samples were maintained the same. Chen *et al*. [18] performed their experiments on their CRISPEY dataset and applied Synthetic Minority Over-Sampling Technique [19] to generate synthetic positive samples and trained Logistic Regression, SVM, Random Forest and Neural Network models. Zhang *et al*. [20] used the scores of score-based methods along with biological properties as features for training an Adaboost [21] model.

Over time, the dataset for Off-Target prediction has grown significantly, and recently most of the works have focused on deep learning for predicting Off-Target sites as traditional machine learning models do not scale well for large datasets [22]. Lin *et al*. [12] presented a convolutional neural network (CNN) based model CNN_Std trained on CRISPOR [23] dataset. They encoded the sgRNA and targeted DNA sequences with the one hot encoding method and superposed (logically ORed) those encodings. A similar encoding approach was followed by other studies, too [12, 24]. Chuai *et al*. [24] produced their own dataset (DeepCRISPR), which has been used by most of the later works as a benchmark dataset. The authors pretrained a Deep Convolutionary Denoising Neural Network based autoencoder using the whole human genome sequence containing almost 0.68 billion unlabeled samples. They used epigenetic features and sequence data to train their CNN model.

Natural language processing (NLP) based techniques have also been employed for Off-Target prediction. For example, Liu *et al*. [25] applied transformers [26] for Off-Target prediction in their proposed tool AttnToMismtach_CNN. Recently, Guan *et al*. [27] have proposed a transformer-based model with a new encoding scheme and they have also designed loss functions to tackle the noise issue in the Off-Target datasets. Chen *et al*. [28] pretrained a BERT [29] model specifically targeting the Off-Target prediction task and used the BERT model’s embedding as features and along with other engineered features for training a LightGBM model [30]. Liu *et al*. [31] proposed CnnCrispr, where they introduced GloVe (Global vectors for word representation) embedding [32] for the input sequences. Yan *et al*. [33] benchmarked various datasets and Off-Target prediction tools and found that structural and energy-based approaches tend to perform better than learning-based methods. However, this finding might just be outdated with the proliferation of new and improved deep-learning-based algorithms [27, 31, 34–36], since this study (more on this in the Discussions Section).

The studies mentioned so far consider only substitution-type mismatches, which is the common cause of Off-Target and the focus of this study. However, a few studies have also considered insertion and deletion-based mismatches between sgRNA and target DNA [34–36]. Here, the work of Störtz *et al*. [35], which we came across during the manuscript writing phase of our research work, deserves some attention. Their model, namely, piCRISPR, utilized the context regions near the target DNA for feature engineering and used a larger dataset for training. However, piCRISPR is incomparable with our work as we only focus on substitution-type mismatches, as mentioned earlier.

Though the application of deep learning has improved the accuracy of Off-Target prediction, the datasets of Off-Target prediction are highly imbalanced, and thus most of the models suffer from precision-recall trade-offs. Another shortcoming of the existing studies is the lack of focus on interpreting the models. A few traditional machine learning models applied feature rankings [13, 37, 38] for On-Target and Off-Target prediction, whereas most of the deep learning models do not provide any interpretation at all. A few deep learning models provide brief interpretation with saliency map [24] or input perturbation-based feature ranking [25] but they do not explore the complex decision-making process with a in-depth analysis. Due to the sensitivity of the application of CRISPR in real life, it is absolutely necessary to understand the inner workings and decision-making process of the prediction models.

In this study, we have attempted to address existing issues in Off-Target prediction. This paper makes the following key contributions.

- We present CRISPR-DIPOFF suite, a set of interpretable deep learning models capable of accurately predicting Off-Target sites using only sequence data (Section 2).
- In parallel, we also present a generalized framework utilizing genetic algorithms that enables the optimization of hyperparameters for deep learning models (Section 3.1). CRISPR-DIPOFF utilizes this framework to achieve improved performance.
- CRISPR-DIPOFF has been effectively interpreted using integrated gradients [39], establishing links with established biological hypotheses (Section 4). Specially our study has identified two possible sub-regions in the seed region, one of which shows positive correlations with Off-Target effects.
- Finally, to facilitate replication and extension of the proposed algorithms, a modular implementation has been developed and has been made available (https://github.com/tzpranto/CRISPR-DIPOFF).
- To the best of our knowledge, our model stands out as the first of its kind, skillfully striking the balance between the precision-recall trade-off while also maintaining high efficacy and interpretability.

## METHODS

### Dataset

The dataset used in the DeepCRISPR [24] study serves as a foundational resource for our analysis and it has been used as the benchmark dataset in most of the previous studies too. It comprises a collection of 30 sgRNA sequences from two cell lines, k562 (12 sgRNAs) and hek293 (18 sgRNAs), along with their corresponding potential Off-Target sites. The authors collected 153 233 loci across the whole genome using a tool named Bowtie 2 [40], allowing up to six mismatches. The dataset was labeled using various whole genome Off-Target detection methods [8, 14, 16, 41, 42]. The dataset is highly imbalanced with a 1:230 ratio of positive and negative samples.

For our experiments, we split the dataset into three subsets: training (60%), validation (20%) and test (20%). The purpose of these subsets is to train the model, tune hyperparameters and evaluate its performance, respectively.

We also have used the GRCh38 (Genome Reference Consortium Human Build 38) dataset the details of which is provided in Section B of the supplementary file. This dataset is used for pretraining purposes in our small-scale experiments using a transformer-based ELECTRA [43] model.

### Performance metrics

For performance evaluation, we have employed various performance metrics like accuracy, precision, recall, F-1 score, Area Under the Receiver Operating Characteristics (AUROC) and Area Under the Precision-Recall Curve (AUPRC). Most of the previous studies reported and compared their results in terms of AUROC score. But AUPRC is often considered a better measure for imbalanced datasets compared with AUROC [25, 44]. Hence in our study, we have used AUPRC as the primary metric for comparison.

### Recurrent neural network-based approach

The Off-Target prediction task involves analyzing two input sequences: sgRNA and target DNA. The objective is to determine the likelihood of potential Off-Target sites. To achieve this objective, we were motivated to explore recurrent neural networks (RNNs) and their variants. RNNs have gained significant popularity and success in sequence prediction tasks across various domains, such as NLP, speech recognition, time series analysis and genomics. While previous works have employed Long Short-Term Memory (LSTM) [20, 25], it has typically been used as a component within a larger model with heterogeneous components. We explored different variants of RNN, like vanilla RNN, LSTM and GRU (Gated Recurrent Unit), and their different compositions such as Bi-directional RNN and Stacked RNN.

#### Data preprocessing

**Figure 1:**
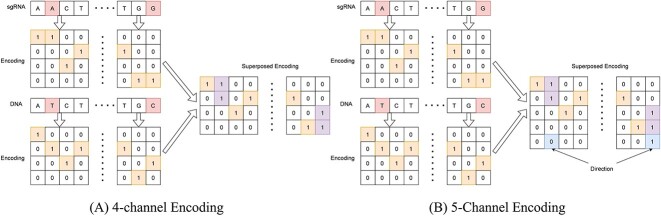
Example of One-hot Encoding. (A) The sgRNA and DNA sequence are encoded with four channels independently and then superposed using logical OR operation. (B) An additional channel has been added for direction.

First, we preprocessed the raw sgRNA and target DNA sequences using two one-hot encoding methods (4-channel and 5-channel) as shown in Figure 1. We followed the same approach used by CNN_Std [12]. For, 4-channel encoding, we converted both sgRNA and DNA sequences to one hot encoded matrix independently and superposed them using logical OR operation to produce the final encoding. We added an additional directional channel in the 5-channel encoding method. In our encoding scheme, the precedence of encoding is ‘A’, ‘T’, ‘C’ and ‘G’. In case of a mismatch, if the higher precedence nucleotide originates from the target DNA sequence, the direction channel is set to ‘1’.

#### Generic model design

**Figure 2:**
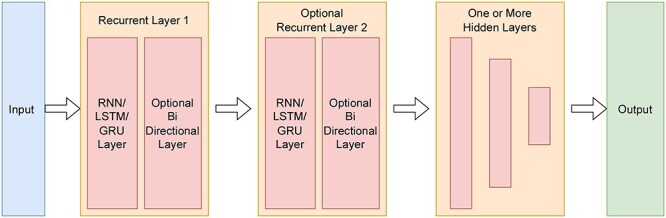
Block Diagram of Generic RNN Based Model. The encoded input matrix passes through one or two uni or bi-directional Recurrent layers, followed by a series of hidden layers. There are ReLU activation and dropout layers in between the hidden layers.

We designed a generic parameterized RNN model which would ease the process of hyperparameter tuning. A simplified block diagram of the model is shown in Figure 2. The model’s input would be the 4/5-channel encoded matrix. The input would then be fed to a recurrent layer(s). Based on the parameter there could be one or two recurrent layers and each layer could be bidirectional or unidirectional. The output from the recurrent layer is passed through a series of fully connected hidden layers each of which gradually halves the size of its previous layer. Finally, there is an output layer. We used CrossEntropy as the loss function and Adam (Adaptive moment estimation) [45] as the optimizer. All the hidden layers were followed by the ReLU activation layer and a dropout layer to tackle overfitting during training.

#### Model selection with genetic algorithms

In order to find the best set of hyperparameters for our model we employed genetic algorithms which are optimization algorithms inspired by the process of natural evolution. Most of the previous works performed model selection only on a limited scale and that too was mostly based on trial and error or adding or removing different components of the model [12, 31]. We selected the eight hyperparameters shown in Table 1 to be tuned for our generic model. Many of the parameters take on continuous values. But due to our limitation of computational resources we prepared a list of possible values for each parameter to be optimized which were reflected in general practice and findings from different empirical studies [46].

**Table 1:** List of hyperparameters and their possible values used in our study

**Hyperparameter Name**	**Range/ Values**	**Step Size for Range**
Batch Size	32–256	Doubled each step
Epochs	10–100	10
Number of Recurrent Layers	1,2	–
Bi-directional Recurrent Layer	True, False	–
Dropout Probability	0.1–0.5	0.05
Hidden Size	32–512	Doubled each step
Number of Hidden Layers	0–6	1
Learning Rate	$1 \times 10^{-5}$ –0.5	Multiple of 5, 10, 50, 100, etc. of the base value

Among the hyperparameters, the possible values for the number of hidden layers are dependent on the hidden size of the recurrent unit. Even without including this hyperparameter, there would be 72 000 possible combinations of the hyperparameters. Training each model on our computational environment took around 10 to 30 min based on the selected hyperparameters. If we were to exhaustively search each combination, it would take about 1000 days! Hence we choose to apply genetic algorithm to intelligently explore the search space. The steps of the genetic algorithm are discussed below.

- **Initial Set of Model Parameters**: First, we prepared a set of hyperparameters by randomly selecting from the possible values for each of the initial models (population).
- **Model Training**: This is the bottleneck step of the whole process where each model is trained with chosen hyperparameters using the training set. In order to tackle the data imbalance during training, we applied a bootstrapping approach where we kept the number of positive and negative samples same in a mini-batch [24].
- **Fitness Assessment**: After the training process, each model’s performance is evaluated using the validation set. AUPRC was chosen as the objective function. We ranked the models of each generation based on the AUPRC score.
- **Model Selection**: We explored two variants of genetic algorithm. In the plain genetic algorithm variant, new set of model hyperparameters are generated using the hyperparameters of the last generation, and the previous generation’s hyperparameters are discarded. This actually causes the already found ‘useful’ models to be lost in the selection process. In another variant, popularly known as Elitist Genetic Algorithm, we selected a certain number of models (Elites) with higher fitness scores which were passed on to the next generation along with the new set of models.
- **Crossover of Model Parameters**: In order to generate a new set of hyperparameters, we first selected models by pairs through tournament selection [47]. Then we performed crossover between the hyperparameters of the models, which is the process of iteratively going through the hyperparameters and for each hyperparameter, with a certain probability, values were swapped between the models. This pairwise crossover process continued until the required number of new hyperparameter sets had been found.
- **Mutation of Model Parameters**: In this process, each set of hyperparameters from crossover is considered one by one. A few hyperparameters with a specified probability are subjected to mutation where their values are substituted with randomly selected values from the possible set of values.
- **New Set of Model Parameters**: After the mutation process, we were left with a new set of hyperparameters. Models with these parameters were trained and the steps continued iteratively until a certain number of generations have passed. In the end, the process returned the best-performing model’s hyperparameters.

In our study, the population, elite and tournament size were 20, 4 and 2, respectively. The crossover and mutation probabilities were 0.3 and 0.2. The genetic algorithm was run for 20 generations. Each of the RNN, LSTM and GRU models works a bit differently so it was not conducive to performing crossover among different types of recurrent networks. Hence we run the genetic algorithm and its elitist variant for each of the recurrent model types independently.

### Transformer-based approach

In recent years, transformer-based large language models (LLMs) like BERT [29] and ELECTRA [43] have revolutionized NLP tasks. We performed small-scale experiments with ELECTRA by pretraining it with unlabeled samples obtained from the GRCh38 dataset which is a comprehensive assembly of the human genome [48]. The pretraining data preparation process was similar to DeepCRISPR [24]. The pretrained models were finetuned with the labeled Off-Target dataset. The details of this approach is provided in Section B of the supplementary file.

### Code, environment and availability

All the deep learning models were designed using the PyTorch deep learning library [49]. The RNN-based experiments including the hyperparameter tuning with genetic algorithm were performed on an Nvidia RTX3090 GPU with 24GB VRAM. The ELECTRA models were pretrained and finetuned on an Nvidia Titan X GPU with 12 GB of VRAM. We used PyTorch Captum [50] library for model interpretation which provides the implementation of integrated gradients. The dataset and source codes are available at https://github.com/tzpranto/CRISPR-DIPOFF.

## RESULTS

### Model selection

In model selection for RNN-based models, we ranked and selected the models based on their AUPRC score on the validation set across all the experiments. The best RNN, LSTM and GRU model’s hyperparameters along with their performance on validation and test set are shown in Table 2. The detailed results of the individual experiments are presented in Section A of the supplementary file.

**Table 2:** Hyperparameters of best RNN, LSTM and GRU models along with their AUPRC score on validation and test

**Model Type**	**Hidden Size**	**LSTM Layers**	**Bi LSTM**	**Hidden Layers**	**Dropout Probability**	**Batch Size**	**Epochs**	**Learning Rate**	**AUPRC on Validation Set**	**AUPRC on Test Set**
**RNN**	256	2	TRUE	0	0.4	256	60	0.00100	**0.7643**	0.5711
**LSTM**	512	1	TRUE	2	0.4	64	50	0.00010	0.7403	**0.7208**
**GRU**	128	2	TRUE	0	0.1	64	30	0.00050	0.7427	0.6859

According to the results, all the models have performed quite well on the validation set in terms of the AUPRC score. We observed that most of the models from the plain genetic algorithm were already found on iterations 0, 1 or 2. This indicates that the best-performing models from the initial generation were not preserved in the subsequent generations and some useful parameter values might have got lost. This effect was reduced using the elitist version of the genetic algorithm which had produced the best-performing RNN and LSTM models. Surprisingly, in both versions of genetic algorithms, the performance of 5-channel encoded data was worse.

The selected best models were trained on the combined training and validation set and their performances were measured on the test set for further comparison with previous studies. The AUPRC scores on the test set have decreased compared with the AUPRC score on the validation set and it is more prominent for the RNN model (rightmost two columns of Table 2). In general, LSTM and GRU can generally store long-term dependency and capture complex patterns compared with RNN [51].

### Comparison with previous studies

**Figure 3:**
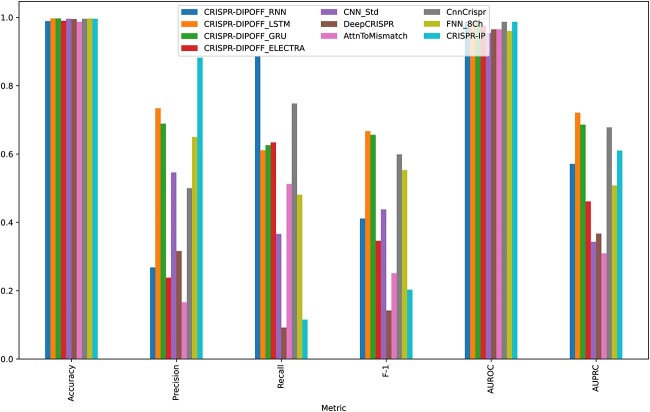
Comparison of different models based on different performance metrics. The accuracy and AUROC score are quite similar for all the models. Other metrics have varied a lot. Our LSTM model achieved the most balanced score with the highest F-1 and AUPRC scores. The underlying values are reported in Table 8 of the supplementary file.

We retrained the deep learning models from previous studies whose source code is publicly available among the models discussed in Section 1. Only the DeepCRISPR [24] model could not be retrained due to the unavailability of source code and data for pretraining but the published model was already trained with the whole DeepCRISPR dataset. All the models were evaluated with the test set and compared with our best-performing RNN, LSTM, GRU and ELECTRA models. In the case of ELECTRA model, we have reported the results of the best finetuned model obtained from our preliminary experiments described in Section B of the supplementary file. The results are shown in Figure 3. The underlying values are reported in Table 8 of the supplementary file.

Both our LSTM and GRU models outperformed all other models in terms of AUPRC score. Although our RNN model showed a slight decrease in performance on the test set, it still performed better than some of the previous models. Contrary to our expectations, the finetuned ELECTRA model did not yield the desired results. Among the baseline models, CnnCrispr demonstrated the most balanced performance, followed by CRISPR-IP. Other baseline models, such as CNN_Std, DeepCRISPR, AttnToMismtach_CNN, FNN_8Ch, demonstrated varying levels of performance.

As the dataset is highly imbalanced toward the negative class, it is expected that accuracy would be high even if a model is bad at predicting the positive class. Hence we observe that all the models have quite similar accuracy. The highest accuracy is 0.997 achieved by LSTM, GRU and the FFN_8Ch model. We can observe the same trend for the AUROC score too. Here the best score 0f 0.991 is achieved by our GRU model. We observe varying scores across the other performance metrics. There is a clear precision-recall trade-off among the models. For example, the CRISPR_IP model has the highest precision (0.882) but the 2nd lowest recall. On the other hand, our RNN model has the highest recall (0.886) but quite low precision. This trade-off is more prevalent in the baseline models and is evident in F-1 scores which are the harmonic mean of precision and recall. Both our LSTM and GRU models have relatively balanced precision and recall with the precision being slightly higher. Hence these two models have the highest F1 score of 0.667 and 0.656, respectively. The other performance metric is AUPRC which is considered a better indicator of performance for highly imbalanced datasets. The highest AUPRC score (0.721) was achieved by our LSTM model and it is followed by our GRU model. The LSTM model has achieved a 6.34% increase in AUPRC score and an 11.35% increase in F-1 score as compared with CnnCrispr, which is currently the best baseline model. Overall, the RNN models, particularly LSTM and GRU, significantly outperformed the baselines. This indicates the superiority of sequence-based models and the potential of hyperparameter-tuned models for improved performance in CRISPR Off-Target prediction. Another important observation is the simplicity of the high-performing models. Further elaborations along this line are done in Section 4.

During the first review cycle of this manuscript, we came across CrisprDNT [27], which is a deep learning model consisting of CNN, Bi-LSTM and Transformers for Off-Target prediction focusing on a new encoding scheme and custom loss function to tackle noise in the datasets. Though the authors have performed experiments on various datasets, many of these have overlaps with the DeepCRISPR dataset [24] used in our work. Our preliminary comparison between CRISPR-DIPOFF and CrisprDNT shows a better F1-score for the former (0.667 versus 0.652) with CRISPR-DIPOFF showing a more balanced precision-recall tradeoff: precision and recall of CRISPR-DIPOFF (CrisprDNT) are 0.734 (0.758) and 0.611 (0.573), respectively. In this connection, the much simpler architecture of CRISPR-DIPOFF is worth recalling.

Interestingly, CrisprDNT was tested on the DeepCRISPR dataset (as an independent dataset) and reported to show good generalization. While this sounds promising, the overlap between the DeepCRISPR dataset and the dataset used to train CrisprDNT suggests a data leakage, thereby reducing the appeal of this result. Nevertheless, irrespective of the results, the novel encoding scheme applied by the authors could be explored in the future for further improvement of our method.

## DISCUSSIONS

Some interesting and insightful observations may be derived from the results and analysis conducted in our experiments. Firstly, the ‘intelligent’ search mechanism for hyperparameter tuning through the use of genetic algorithms has yielded models with superior performance, despite the model having simple architecture. This observation highlights two important insights. One is the emphasis on the criticality of proper hyperparameter tuning in achieving superior performance. By carefully selecting and tuning the hyperparameters, we apparently were able to unlock the full potential of the RNN-type models and surpass the performance of the more intricate and complex baseline models. The other insight aligns with the principle of Occam’s Razor, which suggests that simpler models are often more likely to be correct among competing hypotheses. In the context of our study, this implies that a well-optimized and properly tuned simpler RNN-based architecture can effectively capture the underlying patterns and dependencies in CRISPR Cas-9 Off-target prediction better than more complex models.

Secondly, among the different encoding schemes used, the 4-channel encoding approach has consistently produced the best results for the RNN, LSTM and GRU models. This finding suggests that the addition of the directional feature in 5-channel encoding may have interfered with the generalization capability of the RNN models. Alternatively, it is possible that the specific mismatch patterns themselves, rather than the direction of the mismatches, play a more significant role in determining Off-Target prediction. Further analysis is needed to better understand the underlying mechanisms driving the performance variations observed across different encoding schemes.

Additionally, the finetuned ELECTRA model did not yield the expected output, which can be attributed to the limited scale pretraining. Despite its below-par performance, we find it worth discussing as a second independent (computational) validation for our interesting observation obtained from the LSTM model’s interpretation, as discussed in the following section.

### Model interpretation

We have applied integrated gradients [39], an axiomatic interpretation technique, for explaining the predictions of our deep learning models, which quantifies the impact of each feature by computing the accumulated gradients along the integration path from a baseline input to the actual input.

### Interpretation of the LSTM model

**Figure 4:**
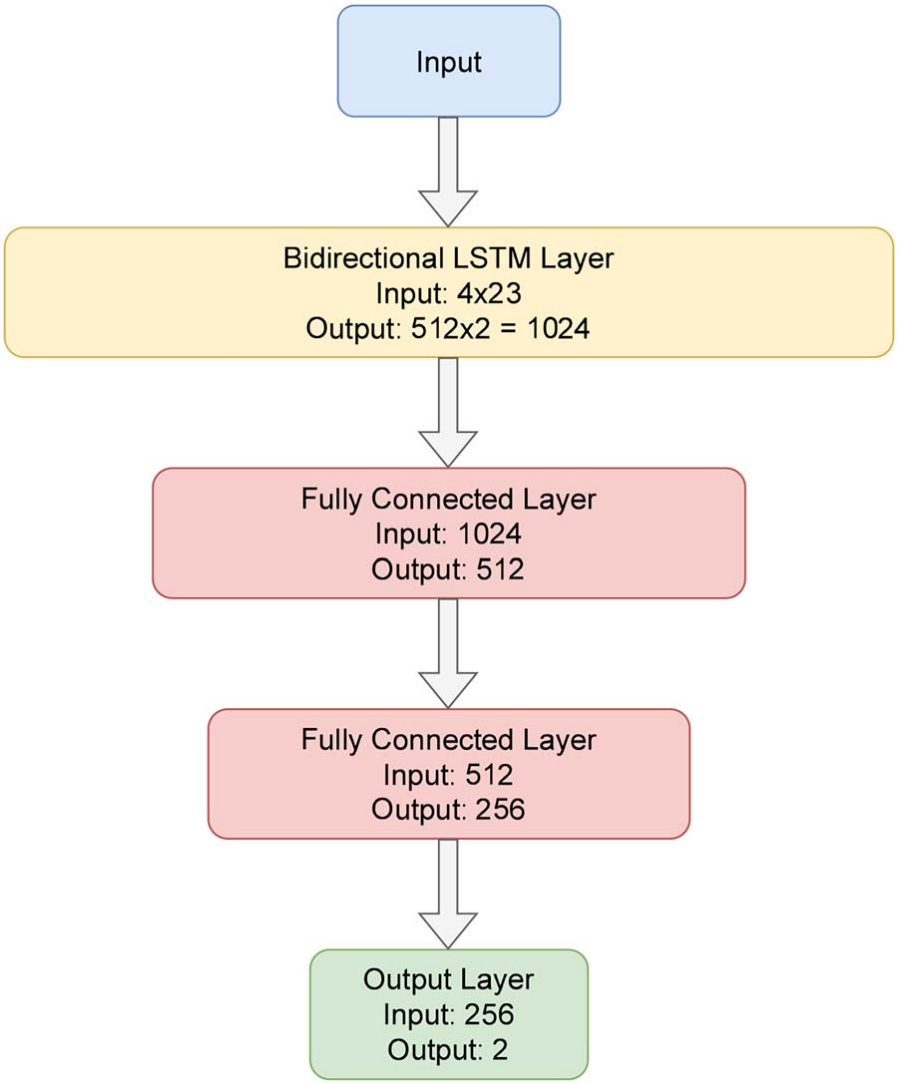
Architecture of best-performing LSTM Model. The model has a bidirectional LSTM followed by two hidden layers and an output layer.

In our study, the best-performing model is an LSTM model which is trained on the 4-channel encoded input. This model’s architecture is shown in Figure 4. The model has a bidirectional LSTM layer followed by two hidden layers and an output layer. The hidden layers have 512 and 256 neurons, respectively.

#### Feature importance

**Figure 5:**
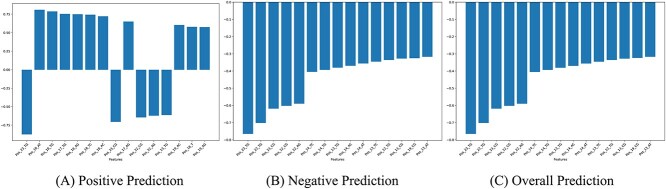
Top 15 features contributing most to (A) positive, (B) negative and (C) overall prediction with respect to positive class.

We calculated attribution scores for all the test samples using Integrated Gradients with respect to the positive class. For each sample, we normalized the attribution scores for all the features present in that sample. then we averaged the score of each feature across all the samples. Based on this score, the top 15 contributing features for positive, negative and overall prediction of the model are shown in Figure 5. Due to the higher ratio of negative samples, the most contributing features for negative and overall prediction are almost the same. We have drawn a few major observations from the importance scores as follows.

- **The effects of Mismatch**: All the top contributing features are related to mismatches. This is intuitive as mismatches are the main reason for Off-Target. We found that among the top 50 features, the number mismatch type features for the positive and negative classes are 38 and 44, respectively.
- **Different Regions in sgRNA**: The 23-mer sequence can be divided into three main regions: PAM (position 21-23), seed or PAM proximal (5 to 12 nucleotides upstream or near the PAM sequence) and PAM distal (rest of the nucleotides) [52]. The 21st nucleotide is a wildcard nucleotide (N), and hence we can observe that none of our top contributing features indicate mismatch at the 21st nucleotide. The other two nucleotides of PAM contain ‘GG’ for Cas-9, and we observe that mismatch with ‘G’ at these positions heavily contributes negatively to predictions. The mismatch features at Positions 17, 18 and 19 have a strong positive correlation with Off-Target. Other top-ranked features have negative correlation scores and they are at positions 12 to 14.
- **Sub-regions in the Seed Region**: The total number of positively correlated mismatch features for positive prediction is 45, and among them, half are at the PAM proximal region (positions 16 to 20) and the rest are at the PAM distal region (positions 1 to 10). The mismatches at positions 11 to 15 have a negative correlation. On the other hand, for negative and overall prediction, only 12 mismatch-type features have a positive correlation and 11 of them are at the PAM proximal region (positions 17 to 20) and the other one is at the PAM distal region. Previous studies have identified that mismatches close to PAM, especially in the so-called seed region, decrease the likelihood of Off-Target effects, and mismatches at the PAM distal region are sometimes tolerated during sgRNA-DNA binding, which results in Off-Target effect [8, 11, 53]. But our model’s interpretation further suggests quite strongly that there are probably two separate portions in the seed regions. One portion is closer to the PAM (positions 16/17 to 20), and the other is adjacent to the PAM distal region (positions 11 to 15/16). According to Zheng *et al*. [54], there is a core region inside the seed region, and the mismatch therein strongly affects the cleavage activity of CRISPR. The 2nd portion of the seed region detected in our study closely overlaps with the core region. It is possible that the deep learning model has learned to distinguish certain features in a manner that somewhat extends the established hypothesis.
- **Mismatch containing ‘TG’ and ‘CG’**: A closer observation of the top-ranked features reveals that mismatches are dominated by ‘TG’ and ‘CG’ type substitutions. This was also reported in DeepCRISPR [24] as a distinguishable property.

#### Layer importance

**Figure 6:**
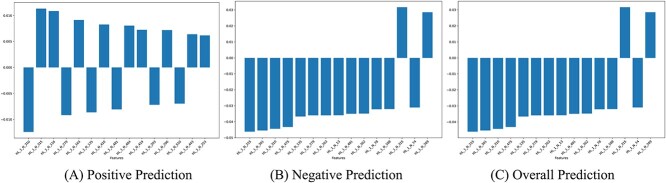
Top 15 neurons contributing most to (A) positive, (B) negative and (C) overall prediction with respect to positive class.

Layer attributions calculated with integrated gradients were used to interpret layer importance. It represents the significance of all the neurons contributing to the output of a specific layer. For each test sample, the attribution score of each neuron of each layer was calculated and averaged across positive, negative and all samples. Our LSTM model has two hidden layers with 512 and 256 neurons, respectively. The top 15 contributing neurons on positive, negative and overall predictions are shown in Figure 6.

**Figure 7:**
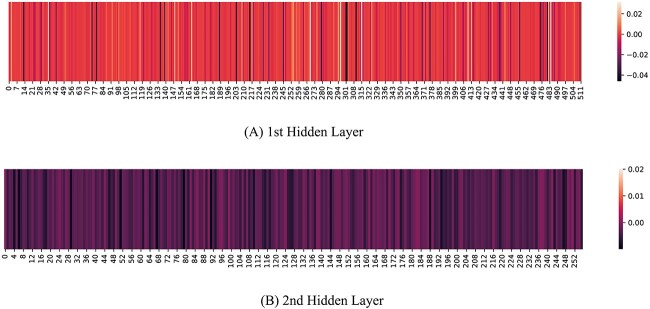
Activation heatmap of neurons of (A) 1st and (B) 2nd hidden layer.

All the neurons in Figure 6 are from hidden layer 1. In fact, only 16 out of the top 100 neurons in terms of overall prediction are from the 2nd hidden layer. This clearly indicates that the 1st hidden layer has more leverage on the final model output. Being directly fed with the LSTM’s output, the 1st hidden layer is able to capture more generalized context. The dominance of 1st hidden layer over the output is clearly visible from the heatmaps shown in Figure 7. The heatmap of the 1st hidden layer is filled with reddish colors, whereas the 2nd hidden layer contains dark patches denoting minimal activity.

### Interpretation of the ELECTRA model

We only interpreted the embedding layers of the finetuned ELECTRA model (8). Similar to the interpretation of our LSTM model, we observe that the attribution scores indicate four distinguishable regions in the DNA tokens in terms of consecutive positive and negative scores. The regions are PAM, PAM distal (position 1 to 7) and two sub-regions in the seed region. One of the sub-regions (position 16 to 20) has a positive correlation with the Off-Target effect, and the other has a negative correlation. This is consistent with our findings in the LSTM model’s interpretation. This further strengthens our earlier observation that there are two sub-regions in the seed regions and one of them might be tolerable for mismatches.

**Figure 8:**
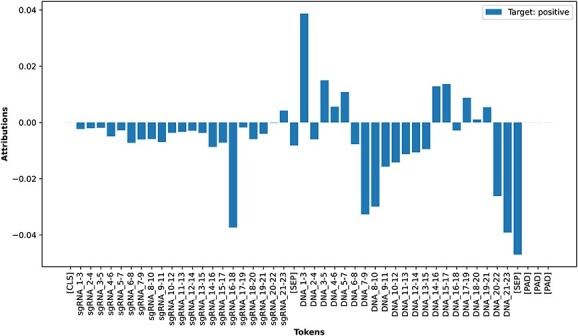
Attribution scores at embedding layers for all the input tokens of finetuned ELECTRA model.

Following some recent literature [38, 55], one of the motivations of this research was to apply only sequence data for the task at hand. This motivation stems from the fact that if we incorporate various other structural information, we usually need to use some other models and while doing so introduce inherent inaccuracies therefrom. Hence, we did not incorporate epigenetic, structural and energy-based features. Informatively, in a benchmarking study, Yan *et al*. [33] found that energy-based models tend to outperform learning-based methods, including DeepCRISPR [24], which attained the highest AUROC and AUPRC score after the energy-based models in their study. However, this finding seems outdated as many deep learning-based models with better efficacy have been published since then (some are considered in out comparative analysis as reported in Figure 3 ). Crucially, in the recently published work of Störtz *et al*. [35], the authors have found less importance in energy-based features compared with sequence-based features. Consequently, we did not include the non-learning-based studies presented in the above benchmarking paper in our comparative analysis.

## CONCLUSION

CRISPR Cas-9 has shown great promise in the field of genome-editing for the past few years but the possibility of Off-Target effects still remains a monumental challenge. The current state-of-the-art deep learning models suffer from precision-recall trade-off and due to the lack of interpretations, these models are still black boxes to the end users. Here we have successfully developed interpretable deep learning models capable of predicting Off-Target sites with high efficacy surpassing the previous studies using only sequence data. Through the interpretation of our models, we seem to have unraveled the existence of sub-regions within the seed region. This can be seen as an extension/elaboration of the current hypothesis and it calls for further biological and computational research.

*Future Extensions:* As an immediate future work, other Cas variants like Cas-12 and Cas-13, insertion–deletion-type mismatches and larger context regions of the target DNA could be explored. Also, more benchmark datasets from different cell types and species could be explored to enhance the applicability and robustness of our proposed methodology. We are thus swayed to conduct a comprehensive benchmark study including more datasets and more models (both learning and non-learning). In addition, proper pretraining of ELECTRA or similar LLMs, particularly on the whole genome sequence, could be used for both On-Target and Off-Target predictions at the same time and even for any other predictive tasks requiring genomic contexts.

*Limitation of this study:* Our study has some limitations. Firstly, as has already been alluded to above, we only considered substitutions which is the common cause of Off-Target and did not consider insertion and deletion-based mismatches. Secondly, we did not consider the structural and energy based models in our comparative study because as per recent evidences, deep learning based models are expected to significantly outperform them. Finally, we experimented with only one dataset. While the DeepCRISPR dataset that we used is a comprehensive dataset, and many of the datasets found in the literature are included therein, we actively will explore more datasets as and when available in the literature.

**Key Points**

- We have presented a set of interpretable deep learning models capable of accurately predicting Off-Target sites using only sequence data.
- We have developed a generalized framework utilizing genetic algorithms that enables the optimization of hyperparameters for deep learning models.
- We have identified two possible sub-regions in the seed region one of which shows positive correlations with Off-Target effects using model interpretation with Integrated Gradients.
- To the best of our knowledge, our model stands out as the first of its kind, skillfully striking the balance between the precision-recall trade-off while also maintaining high efficacy and interpretability.

## Supplementary Material

Supplementary_File_bbad530

## Author Biographies

**Md. Toufikuzzaman** is a Lecturer at the Department of Computer Science and Engineering, Bangladesh University of Engineering and Technology (BUET). His research interests are the application of machine learning, artificial intelligence and bioinformatics.

**Md. Abul Hassan Samee** is an Assistant Professor at the Department of Integrative Physiology, Baylor College of Medicine. His research focuses on biophysics, machine learning and comparative genomics.

**M. Sohel Rahman** is a Professor at the Department of Computer Science and Engineering, Bangladesh University of Engineering and Technology (BUET). His research interest includes algorithms, bioinformatics and metaheurisitics.
