# Supplementary material for: CRISPR-DIPOFF: an interpretable deep learning approach for CRISPR Cas-9 off-target prediction
Source: Brief Bioinform. 2024 Feb 8;25(2):bbad530. doi: 10.1093/bib/bbad530 (PMC10883906; doi:10.1093/bib/bbad530)
Supplement: Supplementary_File_bbad530 [file supplementary_file_bbad530.pdf]

Supplementary File

A. Model Selection with Genetic Algorithm

In order to perform model selection, we ran the plain Genetic Algorithm (GA) and Elitist Genetic Algorithm (EGA) separately for 20 generations and each generation had 20 models. We ran separate experiments for the 4-channel and 5-channel encoding schemes. Including the initial population, we trained a total of 420 models in each experiment. The same experiment was run independently for RNN, LSTM, and GRU-type recurrent networks. As each of these models works a bit differently, it was not conducive to performing crossover among different types of recurrent networks. Hence we could not include model type (RNN, LSTM, or GRU) as another hyperparameter for our tuning algorithm. Notably, instead of checking 72000 (or even more) possible models for each type of recurrent network, we only, but intelligently explored 420 thereof. We ranked the models based on their AUPRC score on the validation set.

The results of the plain genetic algorithm on 4-channel and 5-channel encoded input are shown in Table 1 and Table 2 respectively. We have shown results for the top three models for each recurrent network type. According to the result, all the models have performed quite well on the validation set compared to the baselines in terms of the AUPRC score. But it is interesting to observe that most of the models were already found on iterations 0, 1, or 2. Later generations did not improve the performance. This indicated that the best-performing models from the initial generation were not preserved in the subsequent generations and some useful parameter values might have gotten lost. Hence we used Elitist Genetic Algorithm to preserve the best-performing models. We also draw an interesting observation that most of the best-performing models have hidden size 512.

The experiments using Elitist Genetic Algorithm (EGA) were similar to the plain Genetic Algorithm approach except that we preserved the four best-performing models of the current generation for the next generation. The results of the elitist genetic algorithm on 4-channel and 5-channel encoded input are shown in Table 3 and Table 4 respectively. The tables reveal that the models have gradually improved over subsequent iterations. The performance in terms of AUROC is quite well on 4-channel encoded input. Surprisingly, in both GA and EGA, the performance of 5-channel encoded data is worse. This observation suggests that the mismatch itself might be the primary factor influencing Off-Target prediction, and incorporating the direction of the mismatch as an engineered feature may have introduced unnecessary noise or complexity to the models.

Table 1. Parameters and Results of Best RNN, LSTM, and GRU Models Obtained from Genetic Algorithm with 4-Channel Encoded Input. AUPRC score has been calculated on the Validation set.

Model Type	Iteration	Hidden Size	LSTM Layers	Bi LSTM	Hidden Layers	Dropout Probability	Batch Size	Epochs	Learning Rate	AUPRC
**RNN**	3	512	1	FALSE	2	0.4	256	30	0.00050	0.7080
	2	512	2	TRUE	2	0.1	64	50	0.00005	0.6914
	2	512	1	FALSE	2	0.2	256	20	0.00010	0.6578
**LSTM**	1	512	2	FALSE	2	0.2	64	20	0.00005	0.7324
	0	64	1	TRUE	4	0.15	256	30	0.00100	0.7203
	0	512	2	TRUE	2	0.1	64	50	0.00005	0.7120
**GRU**	0	128	2	TRUE	0	0.1	64	30	0.00050	0.7427
	1	512	2	FALSE	2	0.25	64	20	0.00005	0.7410
	1	512	2	FALSE	2	0.2	64	20	0.00005	0.7016

Table 2. Parameters and Results of Best RNN, LSTM, and GRU Models Obtained from Genetic Algorithm with 5-Channel Encoded Input. AUPRC score has been calculated on the Validation set.

Model Type	Iteration	Hidden Size	LSTM Layers	Bi LSTM	Hidden Layers	Dropout Probability	Batch Size	Epochs	Learning Rate	AUPRC
**RNN**	3	512	**1**	FALSE	2	0.1	32	90	0.00010	0.7135
	**0**	**512**	1	**FALSE**	2	**0.15**	256	30	0.00050	0.7045
	1	128	2	FALSE	**0**	0.1	256	20	0.00100	0.6983
**LSTM**	1	64	2	TRUE	4	0.5	256	30	0.00500	0.7157
	0	64	1	TRUE	4	0.15	256	30	0.00100	0.6890
	2	512	1	FALSE	2	0.4	64	50	0.00050	0.6885
**GRU**	2	512	1	FALSE	2	0.4	64	50	0.00050	0.6885
	3	64	2	FALSE	1	0.1	256	30	0.00100	0.6872
	5	256	1	TRUE	2	0.45	32	50	0.00010	0.6860

Table 3. Parameters and Results of Best RNN, LSTM, and GRU Models Obtained from Elitist Genetic Algorithm with 4-Channel Encoded Input. AUPRC score has been calculated on the Validation set.

Model Type	Iteration	Hidden Size	LSTM Layers	Bi LSTM	Hidden Layers	Dropout Probability	Batch Size	Epochs	Learning Rate	AUPRC
**RNN**	4	256	2	TRUE	0	0.4	256	60	0.00100	0.7643
	3	128	1	FALSE	4	0.2	128	30	0.00100	0.7417
	9	128	1	TRUE	4	0.15	128	30	0.00100	0.7284
**LSTM**	1	512	1	TRUE	2	0.4	64	50	0.00010	0.7403
	7	512	1	TRUE	2	0.1	256	80	0.00005	0.6998
	1	64	1	TRUE	4	0.15	256	30	0.00100	0.6980
**GRU**	1	512	1	FALSE	2	0.4	64	50	0.00010	0.7403
	1	128	1	FALSE	4	0.2	64	50	0.00010	0.7168
	1	512	2	FALSE	2	0.1	64	50	0.00005	0.7079

Table 4. Parameters and Results of Best RNN, LSTM, and GRU Models Obtained from Elitist Genetic Algorithm with 5-Channel Encoded Input. AUPRC score has been calculated on the Validation set.

Model Type	Iteration	Hidden Size	LSTM Layers	Bi LSTM	Hidden Layers	Dropout Probability	Batch Size	Epochs	Learning Rate	AUPRC
**RNN**	1	512	1	FALSE	2	0.15	256	30	0.00050	0.7058
	3	512	2	FALSE	2	0.1	256	80	0.00100	0.6951
	6	512	2	FALSE	2	0.2	256	80	0.00050	0.6754
**LSTM**	3	512	2	FALSE	2	0.1	256	80	0.00100	0.6951
	6	512	2	FALSE	2	0.2	256	80	0.00050	0.6754
	7	512	1	TRUE	2	0.1	256	80	0.00005	0.6266
**GRU**	4	512	2	FALSE	2	0.1	256	80	0.00100	0.6951
	6	512	2	FALSE	2	0.2	256	80	0.00050	0.6754
	7	512	1	TRUE	2	0.1	256	80	0.00005	0.6266

B. Transformer-Based Approach

In recent years, the development of transformer-based models, such as BERT (Bidirectional Encoder Representations from Transformers) and ELECTRA (Efficiently Learning an Encoder that Classifies Token Replacements Accurately), has revolutionized natural language processing tasks. One key aspect that has contributed to the success of these models is pretraining which involves training the models on large-scale datasets, allowing them to learn general language representations. We designed an ELECTRA-based pipeline to utilize large pretrained models for Off-Target prediction. ELECTRA is considered an improvement over BERT due to its adversarial training approach, which leads to better model generalization. It is computationally more efficient, requiring fewer parameters and less pretraining time. ELECTRA also exhibits improved robustness and stability, making it a preferred choice for natural language processing tasks.

Data Preprocessing

We utilized the GRCh38 (Genome Reference Consortium Human Build 38) as the dataset for pretraining which is a comprehensive assembly of the human genome [38] containing autosomes, sex chromosomes, and mitochondrial chromosomes. We collected this dataset from the Ensembl [49] project's repository [2].

We followed a data processing approach similar to that used in DeepCRISPR's pretraining. But we used GRCh38 instead of GRCh37 as the data source for the human genome sequence. We used the Cas-OFFinder tool to find sample pairs that allowed up to six nucleotide mismatches where the first sequence (sgRNA) ends with 'NGG'. This process resulted in a pretraining dataset comprising approximately 317 million samples. In our study, we explored two approaches to construct the vocabulary: overlapping tri-mer and byte pair encoding (BPE). Both approaches have five special tokens: [UNK], [CLS], [SEP], [MASK], and [PAD] representing unknown, classification, separator, mask, and padding tokens respectively. Overlapping tri-mer involves dividing the sequences into overlapping three-nucleotide segments. The other approach, unigram Byte Pair Encoding (BPE), is a subword tokenization technique that involves iteratively merging the most frequent character pairs in a corpus to create a vocabulary of subword units.

After forming the vocabulary we preprocessed the pretraining samples. Each sequence pairs were first tokenized. The tokens of two sequences were separated by a [SEP] token and a [CLS] token was inserted before the first token of sgRNA. Another [SEP] token was added at the end of the target DNA tokens. The maximum sequence length was set to 48 and the remaining positions after the input tokens were filled with [PAD] tokens. An input mask was generated to identify different sequences (0 for sgRNA and 1 for potential target DNA). Attention mask differentiated the actual input tokens from the [PAD] tokens.

Pretraining the ELECTRA model

In the process of pretraining ELECTRA, a subset of approximately 15% of the samples was selected for replacement with a [MASK] token. This masked portion of the data was then passed through a generator, which substituted the masked tokens with generated tokens. The discriminator's task was to determine whether the tokens had been replaced by the generator or not. To accommodate limited resources, we conducted pretraining using two relatively smaller versions of the ELECTRA model, namely, the Tiny and the Small models. The training process consisted of 100,000 steps with a batch size of 128. Towards the end of the training, the loss function stabilized over the last few thousand steps. The two smaller versions of the ELECTRA model employed the parameter configurations shown in Table 5. We kept all other parameters consistent with the official ELECTRA code repository. The tiny model underwent training for approximately 7 days, while the small model was trained for 10 days. Both models were trained separately using tri-mer and byte pair encoded tokens.

Table 5. Parameters for ELECTRA Tiny and Small Model.

Parameter	Tiny Model	Small Model
Embedding Size	128	512
Hidden Size	256	512
Intermediate Size	512	1024
Encoder Layers	6	8
Attention Heads	6	8

Finetuning ELECTRA Model for Off-Target Prediction

Following pretraining, the ELECTRA model was further finetuned on a specific downstream task, where it was trained on the labeled DeepCRISPR dataset. During finetuning, the model's parameters were adjusted to optimize its performance on the task's objective using supervised learning. We conducted experiments with different numbers of layers (ranging from 1 to 3) attached to the output of the [CLS] token of the ELECTRA model and finetuned them. Models pretrained on both tri-mer and byte pair encoded tokens were finetuned separately. Initially, during the finetuning process, the model faced difficulties in learning. It exhibited a tendency to predict all samples as either 1 or 0. To overcome this, we employed a training technique known as gradual unfreezing [16]. This involved initially freezing the weights of the ELECTRA model and updating only the output layer weights for a few iterations. Subsequently, we gradually unfroze the weights of each ELECTRA layer, starting from the layer closest to the output layer in a bottom-up manner. While this approach led to gradual improvement in the performance of the Tiny model, the

[2] `https://ftp.ensembl.org/pub/release-110/fasta/homo_sapiens/dna/`

Small model still exhibited a tendency to predict all samples as either 0 or 1. As a result, we have focused on comparing the results obtained using the Tiny model.

Results of ELECTRA model

Table 6. Results of finetuned ELECTRA models pretrained with Tri-Mer Encoded (TME) input tokens. The result shows how the performance improved over the gradual unfreezing of ELECTRA layers.

Model Type	Unfrozen Layers	Trainable ELECTRA Parameters	Accuracy	Precision	Recall	F1-Score	AUROC	AUPRC
TME with 1 FCL	None	0	0.7888	0.0174	0.8702	0.0340	0.8772	0.0261
	Layer 6	527104	0.8237	0.0219	0.9237	0.0429	0.9377	0.1035
	Layer 5 to 6	1054208	0.8230	0.0219	0.9237	0.0427	0.9452	0.1169
	Layer 4 to 6	1581312	0.8315	0.0240	**0.9695**	0.0469	0.9585	0.1398
	Layer 3 to 6	2108416	0.9394	0.0597	0.8931	0.1120	0.9775	0.3407
	Layer 2 to 6	2635520	0.9782	0.1365	0.7710	0.2319	0.9760	0.4081
	Layer 1 to 6	3162624	0.9897	0.2409	0.6565	0.3525	**0.9795**	0.4236
	All Layers	3194112	**0.9918**	**0.2842**	0.6031	**0.3863**	0.9542	0.4288
TME with 2 FCL	None	0	0.8078	0.0197	0.9008	0.0385	0.9264	0.0657
	Layer 6	527104	0.8077	0.0208	0.9542	0.0407	0.9369	0.1009
	Layer 5 to 6	1054208	0.8244	0.0229	0.9618	0.0447	0.9479	0.1078
	Layer 4 to 6	1581312	0.8243	0.0231	**0.9695**	0.0450	0.9572	0.1161
	Layer 3 to 6	2108416	0.9380	0.0576	0.8779	0.1080	0.9752	0.2846
	Layer 2 to 6	2635520	0.9772	0.1271	0.7405	0.2170	0.9775	0.3596
	Layer 1 to 6	3162624	0.9857	0.1848	0.6870	0.2913	0.9742	0.3951
	All Layers	3194112	0.9898	0.2378	0.6336	0.3458	0.9752	**0.4613**
TME with 3 FCL	None	0	0.8256	0.0220	0.9160	0.0430	0.9385	0.0777
	Layer 6	527104	0.8127	0.0208	0.9313	0.0408	0.9363	0.0980
	Layer 5 to 6	1054208	0.8024	0.0206	**0.9695**	0.0402	0.9435	0.1095
	Layer 4 to 6	1581312	0.7963	0.0203	0.9847	0.0397	0.9576	0.1129
	Layer 3 to 6	2108416	0.9464	0.0660	0.8779	0.1228	0.9757	0.2432
	Layer 2 to 6	2635520	0.9758	0.1225	0.7557	0.2109	0.9756	0.2317
	Layer 1 to 6	3162624	0.9875	0.2033	0.6565	0.3105	0.9688	0.3995
	All Layers	3194112	0.9881	0.2118	0.6565	0.3203	0.9746	0.3896

During the finetuning phase of the ELECTRA models, we employed a strategic approach to enhance its performance. This involved incorporating additional layers, ranging from 1 to 3, including the output layer, specifically designed for classification purposes. By introducing these extra layers, we aimed to capture more intricate patterns and improve the model's ability to make accurate predictions. To finetune the ELECTRA models, we utilized the gradual unfreezing strategy. This technique allows for a controlled update of the model's weights, starting with the output and additional layers and progressively unfreezing the encoder layers closest to it. In our experiments, we conducted separate finetuning procedures on the Tiny models that were pretrained using both Tri-Mer Encoded (TME) and Byte Pair Encoded (BPE) input tokens. The results of these experiments, as shown in Table 6 and Table 7 for the TME and BPE models, respectively, shed light on the impact of these encoding approaches on the model's performance. Notably, we observed a significant performance advantage for the TME models compared to the BPE models. Among the TME models, the one that yielded the best results incorporated two additional layers, an additional hidden layer and an output layer, following the ELECTRA encoder layers.

Though the overall result does not outperform the baseline models and the RNN-based models, we observe that gradual unfreezing improved the performance of the models. Gradual unfreezing provides better results because it helps to mitigate the problem of catastrophic forgetting. Catastrophic forgetting is a phenomenon that occurs when a machine learning model is finetuned on a new task, and it loses its ability to perform well on the original task. While finetuning the ELECTRA model, we are essentially updating the weights of the model to better fit the new task. However, if we update all of the weights at once, it is possible that the model will forget how to perform the original task which is capturing the general context of the sequences. Gradual unfreezing helps to mitigate this problem by unfreezing the layers of the model from the last layer to the first layer. The last layer of the model contains the least general knowledge, so it is the least likely to be affected by finetuning on a new task.

Figure 9 shows the effect of gradual unfreezing on performance metrics for the best-performing ELECTRA model. It shows that most of the metrics have improved gradually except recall. The initial model had the tendency to predict a lot of Off-Targets

Table 7. Results of finetuned ELECTRA models pretrained with Byte Pair Encoded (BPE) input tokens. The result shows how the performance improved over the gradual unfreezing of ELECTA layers.

Model Type	Unfrozen Layers	Trainable ELECTRA Parameters	Accuracy	Precision	Recall	F1-Score	AUROC	AUPRC
BPE with 1 FCL	None	0	0.6538	0.0106	0.8702	0.0210	0.8174	0.0186
	Layer 6	527104	0.7193	0.0130	0.8626	0.0256	0.8767	0.0469
	Layer 5 to 6	1054208	0.7463	0.0145	0.8702	0.0285	0.8892	0.0564
	Layer 4 to 6	1581312	0.8089	0.0183	0.8321	0.0359	0.9053	0.0774
	Layer 3 to 6	2108416	0.8714	0.0257	0.7863	0.0497	0.9234	0.0990
	Layer 2 to 6	2635520	0.9621	0.0620	0.5573	0.1116	0.9282	0.1716
	Layer 1 to 6	3162624	0.9832	0.1203	0.4656	0.1912	0.9236	0.1681
	All Layers	3266816	0.9879	0.1653	0.4504	0.2418	0.9213	0.2321
BPE with 2 FCL	None	0	0.7275	0.0123	0.7939	0.0243	0.8545	0.0288
	Layer 6	527104	0.7377	0.0139	0.8626	0.0273	0.8809	0.0559
	Layer 5 to 6	1054208	0.7583	0.0156	0.8931	0.0306	0.8950	0.0534
	Layer 4 to 6	1581312	0.8244	0.0196	0.8168	0.0382	0.9098	0.0696
	Layer 3 to 6	2108416	0.8890	0.0280	0.7405	0.0539	0.9217	0.1130
	Layer 2 to 6	2635520	0.9610	0.0603	0.5573	0.1088	0.9260	0.1606
	Layer 1 to 6	3162624	0.9784	0.0945	0.4733	0.1576	0.9251	0.1725
	All Layers	3266816	0.9889	0.1745	0.4275	0.2478	0.9220	0.1788
BPE with 3 FCL	None	0	0.7705	0.0142	0.7710	0.0279	0.8671	0.0346
	Layer 6	527104	0.7058	0.0130	0.9084	0.0257	0.8739	0.0482
	Layer 5 to 6	1054208	0.7350	0.0139	0.8702	0.0273	0.8849	0.0586
	Layer 4 to 6	1581312	0.7871	0.0166	0.8397	0.0326	0.9053	0.0749
	Layer 3 to 6	2108416	0.8908	0.0290	0.7557	0.0558	0.9205	0.0985
	Layer 2 to 6	2635520	0.9597	0.0555	0.5267	0.1004	0.9224	0.1360
	Layer 1 to 6	3162624	0.9731	0.0830	0.5267	0.1435	0.9209	0.1141
	All Layers	3266816	0.9864	0.1439	0.4427	0.2172	0.9156	0.1969

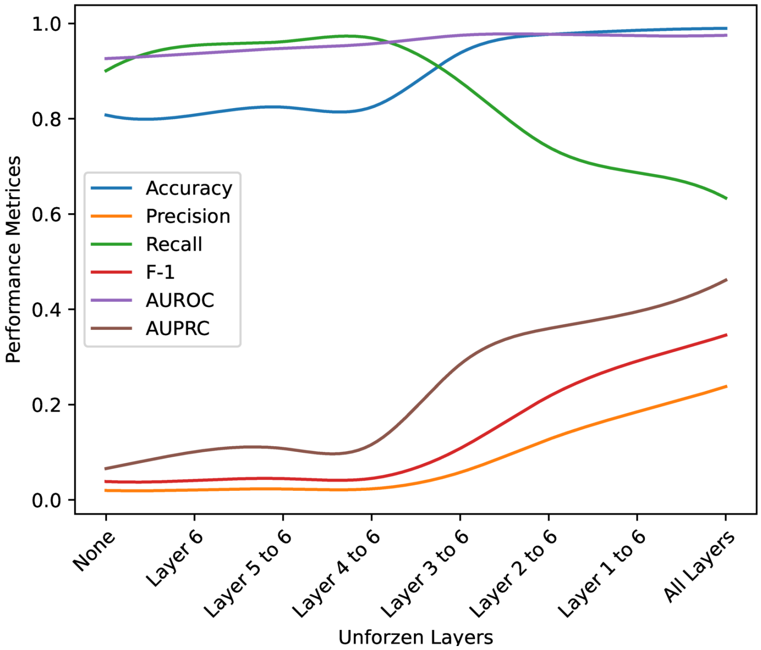

Fig. 9. Effect of gradual unfreezing on different performance metrics. All the performance metrics increased gradually when the parameters of the ELECTRA layers were unfrozen for finetuning one by one. The only exception was recall as it decreased with the increase of precision.

which affected the precision of the model. As the model gradually stroke a balance between precision and recall, precision gradually increased and recall gradually decreased.

Observations

Instead of training the model with the complete human genome, which would have required significant time and resources, we followed the pretraining approach of DeepCRISPR by selecting samples generated by Cas-OFFinder [3]. It resulted in a much smaller pretraining dataset which was manageable within our limited computational resources. However, this limited pretraining dataset may have hindered the generalizability of the model. The overlapping nature of tri-mers during training could also have led the model to easily predict a masked token based on the previous or next token, potentially resulting in earlier convergence and limited generalization. To address these limitations, a more appropriate approach would involve training the ELECTRA model with the entire human genome sequence. This would not only enhance its application for Off-Target prediction but also make it valuable for other tasks related to human genome analysis.

We note however that, despite its below-par performance with respect to some of the baselines and other models in our CRISPR-DIPOFF suite, we find it worth discussing for the following reasons. Firstly, Large Language Models (LLMs) have the potential to propel the advancement of bioinformatics, similar to their impact on NLP. Secondly, despite pretraining ELECTRA in our resource-constrained setting, it showed an indication of possible performance improvement. This suggests that properly pretrained ELECTA or similar LLMs could be Swiss army knife for any biological sequence-related prediction tasks. Finally, the interpretations of the finetuned ELECTRA model acts as the second independent (computational) validation for our interesting observation obtained from the LSTM mode's interpretation as discussed in the following section.

Interpretation of the ELECTRA Model

The ELECTRA model, finetuned for Off-Target prediction, did not perform as expected. Among the different ELECTRA models, the pretrained model on tri-mer encoded tokens and finetuned with two additional layers performed better than others. Despite not meeting our initial performance expectations, we have attempted to interpret the model in a limited capacity. Given the complexity and size of the model, which comprises approximately 3.2 million parameters, interpreting its inner workings becomes an arduous task. To overcome this challenge, we focused our interpretation efforts on the embedding layers of the finetuned ELECTRA model. The ELECTRA model consists of three specific embedding layers for token, token type, and position embedding. We employed the integrated gradients method to calculate attribution scores for these layers. The input tokens used in our experiments are tri-mer encoded sgRNA and DNA sequences, separated by [SEP] tokens. There is also a [CLS] token at the start and a [SEP] token at the end. In order to align with the maximum sequence length, the last three empty positions were filled with [PAD] tokens. Integrated gradients require a baseline sample to compute the gradient along the path. Generally, an input vector containing all zeros is used for that purpose. In the case of ELECTRA, the baseline sample has been prepared with [PAD] tokens.

We computed the average attribution scores for each token across positive, negative, and overall predictions on the finetuned model. To observe the change in the embedding layer we also computed attribution scores for overall predictions on pretrained model (i.e., the model before finetuning). The token importances are illustrated in Figure 10 which clearly demonstrates that the tokens associated with the DNA sequence exhibit significantly higher attribution scores compared to the sgRNA tokens. This observation is well-aligned with the understanding that the sgRNA sequence is engineered, while the target DNA sequence is responsible for introducing mismatches. In practical scenarios, a single sgRNA can potentially interact with numerous off-target DNA sites, and the final determination of off-target effects depends on the mismatches introduced by the DNA sequences. It appears that the embedding layers of the finetuned ELECTRA model have successfully captured this relationship from the training data used during finetuning. The comparison of attribution before and after the finetuning process illustrates how the embedding layer has changed after the finetuning process and has been aware of the biological significance of the specific task of Off-Target prediction.

Similar to the interpretation of our LSTM model, we observe that the attribution scores for negative and overall prediction are almost the same. The attribution scores for overall predictions show some sort of four distinguishable regions in the DNA tokens. The first region is related to PAM and it affects the final prediction negatively. There are two regions with contiguous positive scores. One of them is in the seed region (position 15 to 20) and the other one is in PAM distal region (position 1 to 6). The region in the middle of these two regions contains contiguous negative attributions. This is consistent with our findings in the LSTM model's interpretation, though the regions do not match exactly nucleotide by nucleotide. This further strengthens our observation that there are two sub-regions in the seed regions and one of them might be tolerable for mismatches. Further investigation is required to validate this observation. We anticipate that improving the performance with large-scale pretraining of the ELECTRA model could unlock more complex biological relationships and a deeper understanding of Off-Target effects.

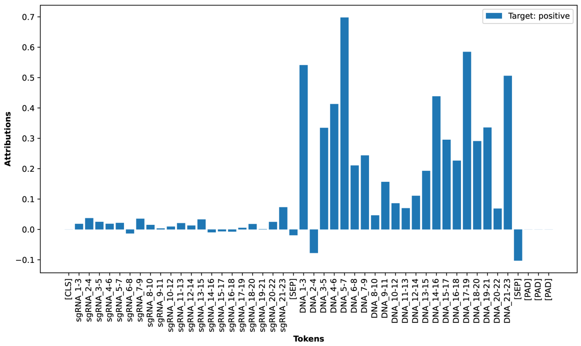

(a) Positive prediction

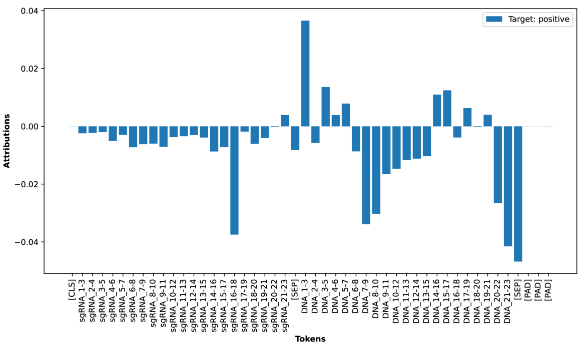

(b) Negative prediction

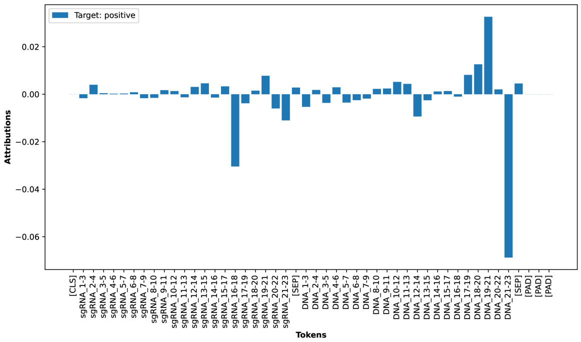

(c) Overall prediction before finetune

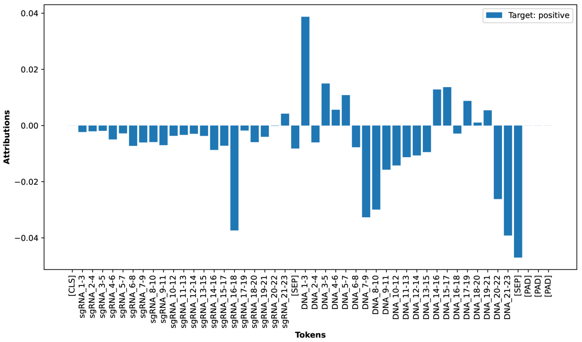

(d) Overall prediction after finetune

Fig. 10. Attribution scores at embedding layers for all the input tokens of finetuned ELECTRA model for (a) positive, (b) negative and, (c) overall prediction before finetune and d) overall prediction after finetune with respect to positive class.

C. Comparison of Performance

A comparison of our models with previous studies is shown in Table 8.

Table 8. Comparison of results of our study with baseline studies. The models of our study have the prefix “CRISPR-DIPOFF”. Our LSTM model has outperformed the models from previous studies by a fair margin.

Model Name	Accuracy	Precision	Recall	F1-Score	AUROC	AUPRC
CRISPR-DIPOFF_RNN	0.989	0.268	**0.886**	0.411	0.987	0.571
CRISPR-DIPOFF_LSTM	**0.997**	0.734	0.611	**0.667**	0.990	**0.721**
CRISPR-DIPOFF_GRU	**0.997**	0.689	0.626	0.656	**0.991**	0.686
CRISPR-DIPOFF_ELECTRA	0.990	0.238	0.634	0.346	0.975	0.461
CNN_Std	0.996	0.546	0.366	0.438	0.954	0.343
DeepCRISPR	0.995	0.316	0.092	0.142	0.965	0.367
AttnToMismtach_CNN	0.987	0.166	0.512	0.251	0.965	0.309
CnnCrispr	0.996	0.500	0.748	0.599	0.987	0.678
FNN_8Ch	**0.997**	0.650	0.481	0.553	0.960	0.508
CRISPR-IP	0.996	**0.882**	0.115	0.203	0.987	0.610
